# Environmental Levels of *para*-Nonylphenol Are Able to Affect Cytokine Secretion in Human Placenta

**DOI:** 10.1289/ehp.0900882

**Published:** 2009-11-23

**Authors:** Nicoletta Bechi, Francesca Ietta, Roberta Romagnoli, Silke Jantra, Marco Cencini, Gianmichele Galassi, Tommaso Serchi, Ilaria Corsi, Silvano Focardi, Luana Paulesu

**Affiliations:** 1 Department of Physiology, University of Siena, Siena, Italy; 2 Obstetrics and Gynecology Division, Hospital, Campostaggia, Siena, Italy; 3 Medical Physics, Department of Physics; 4 Department of Clinical Medicine and Immunological Sciences, Rheumatology Unit and; 5 Department of Environmental Sciences, University of Siena, Siena, Italy

**Keywords:** chorionic villous explants, cytokine network, endocrine disruptors, human placenta, para-nonylphenol

## Abstract

**Background:**

*para*-Nonylphenol (*p*-NP) is a metabolite of alkylphenols widely used in the chemical industry and manufacturing. It accumulates in the environment, where it acts with estrogen-like activity. We previously showed that *p*-NP acts on human placenta by inducing trophoblast differentiation and apoptosis.

**Objective:**

The aim of the present study was to investigate the effect of *p*-NP on cytokine secretion in human placenta.

**Methods:**

*In vitro* cultures of chorionic villous explants from human placenta in the first trimester of pregnancy were treated with *p*-NP (10^−13^, 10^−11^, and 10^−9^ M) in 0.1% ethanol as vehicle. Culture medium was collected after 24 hr and assayed by specific immunoassays for the cytokines granulocyte-macrophage colony-stimulating factor (GM-CSF), interferon-γ (IFN-γ), interleukin (IL)-1β, IL-2, IL-4, IL-5, IL-6, IL-8, IL-10, and tumor necrosis factor-α (TNF-α).

**Results:**

*p*-NP modulated cytokine secretion by inducing the release of GM-CSF, IFN-γ, IL-1β, IL-4, and IL-10, with a maximum effect at 10^−11^ M. It reduced the release of TNF-α at 10^−13^ M, whereas levels of IL-2 and IL-5 remained below the detection limit. IL-6 and IL-8 levels were 100–1,000 times higher than those of other cytokines, and they were not affected by *p*-NP. We observed significant differences from controls (ethanol alone) only for GM-CSF and IL-10.

**Conclusion:**

An unbalanced cytokine network at the maternal–fetal interface may result in implantation failure, pregnancy loss, or other complications. The effects of extremely low doses of *p*-NP on the placental release of cytokines raise considerable concerns about maternal exposure to this endocrine disruptor during pregnancy.

The production and release of chemical substances into the environment continue to increase in modern society. Compounds known as endocrine-disrupting chemicals (EDCs) are a heterogeneous group of contaminants present in the environment and in food that may interfere with the endocrine and reproductive system even at low doses (Kavlock and Ankley 1996). They may bind to hormone receptors, interfering with inhibition of synthesis and/or transport of particular hormones (Neubert 1997).

Alkylphenol ethoxylates, among the most important EDCs, were introduced in the 1940s and are a class of non-ionic surfactants used in detergents, paints, pesticides, personal care products, and plastics (Soto et al. 1991; White et al. 1994). These chemicals are discharged into aquatic environments via urban and industrial wastewaters, where they are broken down biologically to nonylphenol ethoxylate by-products and to the final degradation intermediate nonylphenol (NP) (Rudel et al. 2003; White et al. 1994). These metabolites are also used as intermediates in the chemical industry (Müller and Schlatter 1998).

*para*-Nonylphenol (*p*-NP), a representative alkylphenol widely used in detergents, emulsifiers, and solubilizers, accumulates in the environment, where it acts with estrogen-like activity (de Weert et al. 2008; Ter Veld et al. 2008). Human exposure to *p*-NP may occur by cutaneous absorption, ingestion of contaminated food or water, and inhalation (Guenther et al. 2003; Monteiro-Riviere et al. 2000). Several studies have shown that *p*-NP binds to both estrogen receptor isoforms (ERα, ERβ), competing with the natural estrogen 17β-estradiol (17β-E_2_) (Kwack et al. 2002; Soto et al. 1995; White et al. 1994), although with less potency (Blom et al. 1998; Nagel et al. 1997). Estrogenic activity of *p*-NP has been reported in both *in vitro* studies (Soto et al. 1991; White et al. 1994) and *in vivo* studies (Laws et al. 2000). Induction of estrogen and progesterone receptor synthesis and proliferation of breast cancer cells (MCF-7) have been known since 1991 (Soto et al. 1991), and tumorigenesis in estrogen-sensitive target tissues has been reported in the last 10 years (Blair et al. 2000; Sonnenschein and Soto 1998). Studies in rats have shown that subcutaneous injection of *p*-NP in late pregnancy induces calbindin-D9k (CaBP-9k) mRNA and protein expression in maternal and neonatal uteri, suggesting its potential transfer through the placenta (Hong et al. 2004a, 2004b). CaBP-9k is a cytosolic calcium-binding protein expressed in various tissues (e.g., intestine, uterus, and placenta) and a marker of estrogenic compounds exposure (Choi et al. 2005). Human placenta is an estrogen target tissue expressing both ERα and ERβ (Bechi et al. 2006; Fujimoto et al. 2005; Rama et al. 2004), so it is an interesting model to study the estrogen-like activity of *p*-NP. Using an *in vitro* model of chorionic villous explants, we recently demonstrated that *p*-NP has an estrogen-like activity on first-trimester human placenta, with a higher potency than 17β-E_2_ (Bechi et al. 2006). In particular, exposure to a low concentration of *p*-NP (10^−9^ M) caused an increase of trophoblast differentiation and cell apoptosis.

Placental establishment and development are physiologic processes closely regulated by soluble autocrine/paracrine factors, namely, cytokines (Makrigiannakis and Minas 2007; Robertson et al. 1997; Srivastava et al. 1996). They include interferons (IFNs), interleukins (ILs), colony-stimulating factors (CSFs), tumor necrosis factors (TNFs), chemokines, transforming growth factors, and leukemia inhibitory factors (Vilcek and Le 1994). Many studies conducted in humans and mice have established that a correct balance of cytokines at the maternal–fetal interface is an essential requirement for proper placental development and therefore reproductive success (Chaddha et al. 2004; Chaouat et al. 1990; Cross et al. 1994; Laird et al. 2006; Raghupathy and Kalinka 2008). Alkylphenols, particularly *p*-NP, also act in immune T cells by altering cytokine synthesis in mice (Iwata et al. 2004; Lee et al. 2003, 2004).

The aim of the present study was to investigate whether *in vitro* exposure to low concentrations of *p*-NP (from 10^−13^ to 10^−9^ M), even lower than those found in the environment (Soares et al. 2008), can interfere with placental secretion of cytokines.

## Materials and Methods

### Sample collection

Placental tissues from first-trimester pregnancies (*n* = 6) were obtained at the hospital’s Division of Obstetrics and Gynecology (Campostaggia, Siena, Italy). Only healthy women who underwent elective termination of pregnancy at weeks 7–8 of gestation were included into the study. Written informed consent after full explanation of the purpose of the study was obtained by the physician from each enrolled patient. Gestational age was determined by the date of the last menstrual period and ultrasound measurement of crown–rump length. When there was a discrepancy of ≥ 7 days between the two dating methods, patients were excluded from the study. Tissues were rinsed in cold phosphate-buffered saline (PBS) to remove excessive blood and processed for explant cultures within 2 hr. The present study was approved by the local ethics committee (Siena, May 2004) in accordance with the Helsinki Declaration guidelines.

### Isolation and treatment of chorionic villous explants

Villous explants from placental tissues were dissected as described by Caniggia et al. (1997). Briefly, small fragments of villous tips (15–20 mg wet weight) were placed on Millicell CM culture dish inserts (Millipore Corp., Bedford, MA, USA) previously coated with 180 μL undiluted Matrigel (Collaborative Research, Inc., Bedford, MA, USA) and then inserted in 24-well plates and left overnight at 37°C in a humidified atmosphere of 95% air/5% CO_2_ to allow explant attachment to the Matrigel. After this time, explants were exposed to culture medium containing *p*-NP (Sigma Chemical Co., St. Louis, MO, USA) at 10^−13^, 10^−11^, and 10^−9^ M, or only to vehicle (0.1% ethanol; control cultures) for 24 hr. *p*-NP concentrations and incubation time were selected on the basis of a report by Bechi et al. (2006) showing trophoblast differentiation and cell apoptosis on *p*-NP exposure. The culture medium used was Dulbecco’s modified Eagle’s medium/F12 without phenol red (Gibco, Grand Island, NY, USA) supplemented with 100 U/mL penicillin/streptomycin and 2 mM l-glutamine (Sigma). At 24 hr of incubation, explants were removed from the Matrigel, washed in PBS, frozen, and stored at −80°C until processing for protein extraction. Culture medium was centrifuged at 4°C for 10 min at 10,000 × *g*, divided into aliquots, immediately frozen at −80°C, and maintained frozen until analysis for cytokines and β-human chorionic gonadotropin (β-hCG). In total, six experiments were performed, each using a single placenta. *p*-NP treatments and control cultures were carried out in triplicate, and samples from separate explant cultures were pooled before being processed at the end of incubation.

### Protein extraction

Pooled villous explants from each treatment were homogenized in ice-cold lysing buffer (50 mM Tris-HCl, 50 mM NaCl, 1% Triton X-100, 1% sodium deoxycholate, 0.1% sodium dodecyl sulfate) containing 100 mM sodium orthovanadate and a protease inhibitor cocktail containing 4-(2-aminoethyl benzenesulfonyl fluoride), pepstatin A, E-64, bestatin, leupeptin, and aprotinin (Sigma). Protein lysates were clarified by centrifuging at 13,000 × *g* for 15 min at 4°C. Total protein concentration (mg/mL) was determined by the Quick Start Bradford Protein Assay (Biorad Laboratories, Hercules, CA, USA).

### Multiplex assay for cytokine quantification

Ten cytokines released into the chorionic explant culture medium were measured simultaneously with the Human Ultrasensitive Cytokine 10-Plex Multiplex Bead Immunoassay (Invitrogen, Carlsbad, CA, USA) following the manufacturer’s protocol. The range of detection was 0.75–549 pg/mL for granulocyte-macrophage colony–stimulating factor (GM-CSF), 0.71–515 pg/mL for IFN-γ, 0.38–274 pg/mL for IL-1β, 0.46–333 pg/mL for IL-2, 1.4–1,019 pg/mL for IL-4, 1.26–916 pg/mL for IL-5, 0.56–409 pg/mL for IL-6, 0.65–474 pg/mL for IL-8, 0.45–330 pg/mL for IL-10, and 0.51–369 pg/mL for TNF-α. These values represent the lowest and the highest limit of detection (LOD) for each cytokine, and concentrations outside the LOD were not considered for statistical analysis. We calculated cytokine concentrations in the samples using a standard curve established from serial dilutions of each cytokine standard as described in the manufacturer’s protocol and expressed as picograms per milliliter of culture medium. Cytokine concentrations were normalized to total explant protein content and expressed as picograms per milligram of tissue protein.

### β-hCG assay

The concentration of β-hCG in the explant culture medium was assessed with a commercial immunoenzymometric assay (Radim SpA, Pomezia, Italy) following the manufacturer’s instructions. The limit of sensitivity was 2 mIU/mL and the linear range of detection was 0–2,000 mIU/mL. The concentration was expressed in milli-international units per microgram of total protein in tissue explants.

### Statistical analysis

The effect of *p*-NP on cytokine secretion was expressed as the stimulatory index (SI): the ratio between the cytokine concentration in the culture medium of *p*-NP–treated explant cultures and the corresponding control cultures in each experiment. We used SI for statistical analysis instead of normalized values to examine differences and trends among our data. Analyses were performed with SPSS [version 13 (SPSS Inc., Chicago, IL, USA) for Mac OS X Tiger (version 10.4.11; Apple, Cologno Monzese, MI, Italy)]. The Shapiro–Wilk test and Q-Q plots were used to confirm normality; Levene’s test and detrended Q-Q plots were respectively used to confirm homoskedasticity and the lack of outliers. Statistically significant differences were determined by one-way analysis of variance (*p* ≤ 0.05).

## Results

### Detection of cytokines

We analyzed 24 samples, including three *p*-NP treatments (10^−13^, 10^−11^, 10^−9^ M) and one control (ethanol 0.1%) from six separate experiments, for GM-CSF, IFN-γ, IL-1β, IL-2, IL-4, IL-5, IL-6, IL-8, IL-10, and TNF-α by means of specific immunoassays. We identified GM-CSF, IFN-γ, IL-1β, and IL-4 in all samples analyzed. We detected IL-10 and TNF-α in 83.3% and 50% of all samples, respectively, whereas their levels were less than the respective LODs, < 0.45 pg/mL and < 0.51 pg/mL, in the other samples. IL-2 and IL-5 were below the respective LODs (< 0.46 and < 1.26 pg/mL), whereas IL-6 and IL-8 exceeded the respective LODs, > 409 and > 474 pg/mL, in 100% of the samples (Table 1).

### Cytokines in p-NP–treated cultures

**Figure 1:**
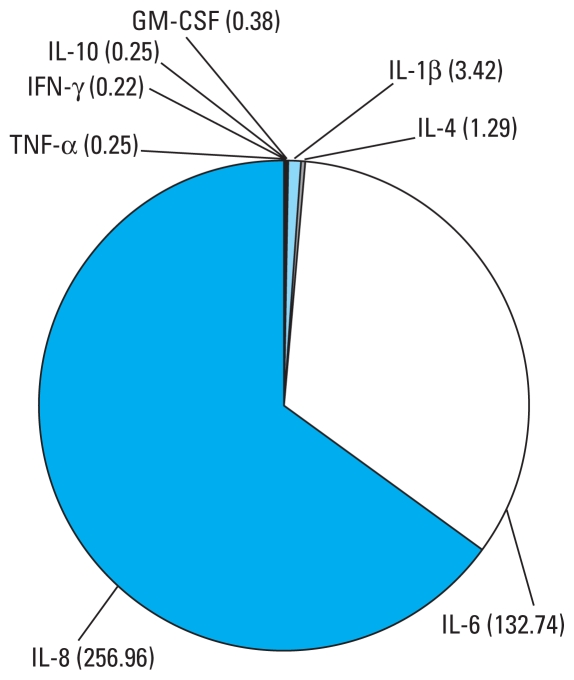
Representative pie chart of cytokine concentrations in the culture medium of chorionic villous explants from human placenta at the first trimester of pregnancy exposed to 0.1% ethanol for 24 hr (control cultures). Numbers in parentheses are cytokine concentrations (pg/mg total tissue protein).

Control cultures of first-trimester human placenta exposed only to vehicle (0.1% ethanol) released GM-CSF, IFN-γ, IL-1β, IL-4, IL-6, IL-8, IL-10, and TNF-α into the culture medium (concentrations and interindividual variances reported in Table 1). Because the levels of IL-6 and IL-8 were above the LOD in all samples examined, we determined the concentrations of these cytokines in a separate assay running together pure and 1:10 diluted samples from a representative experiment. The concentrations of IL-6 and IL-8 were 100–1,000 times higher than those of the other cytokines (Figure 1).

**Figure 2:**
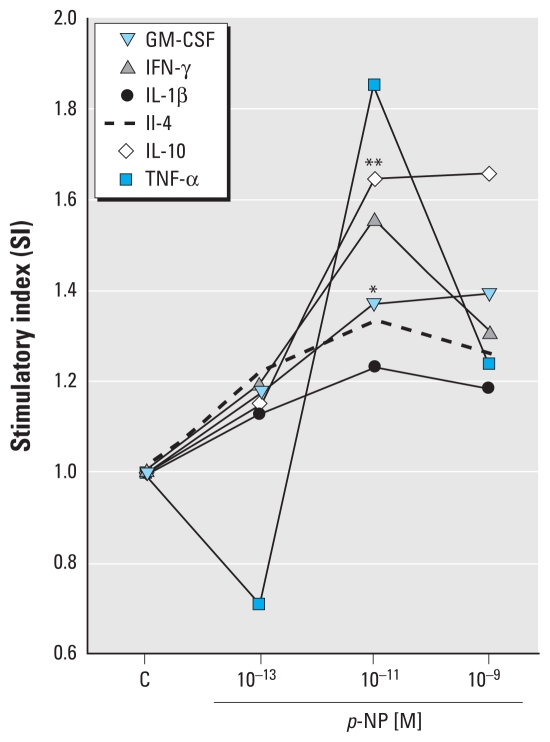
Cytokine secretion in the culture medium of chorionic villous explants from first-trimester placenta exposed to *p*-NP at various concentrations for 24 hr. SI is the ratio between the cytokine concentration in culture medium of *p*-NP–treated explant cultures and the corresponding control cultures (C) in each experiment. Data are the mean of experiments including detectable amounts of cytokines (*n* = 6 for GM-CSF, IFN-γ, IL-1β, and IL-4; *n* = 5 for IL-10; *n* = 3 for TNF-α). A significant increase was observed for GM-CSF and IL-10 (*p = 0.045 and **p = 0.011, respectively).

Treatment with *p*-NP for 24 hr increased the secretion of GM-CSF, IFN-γ, IL-1β, IL-4, and IL-10 with respect to control cultures (Figure 2). We observed a biphasic effect of *p*-NP for TNF-α: It was suppressive at the lowest concentration (10^−13^ M) and stimulatory at the others, with a peak at 10^−11^ M (Figure 2). To reduce the subject/tissue variability, we expressed data as the mean SI [ratio between the cytokine concentration in culture medium of treated explant cultures and the corresponding untreated cultures (0.1% ethanol) in each experiment]. We observed increasing SIs for GM-CSF, IFN-γ, IL-1β, IL-4, TNF-α, and IL-10, with a maximum effect at 10^−9^ M *p*-NP for GM-CSF and at 10^−11^ M *p*-NP for the other cytokines (Figure 2). The increase was significant for GM-CSF and IL-10 (*p* = 0.045 and *p* = 0.011, respectively) at 10^−11^ M. IL-2 and IL-5 remained below the LOD (data not shown), whereas IL-6 and IL-8 were above the LOD and we determined their concentrations in a representative experiment as described for the control cultures. We observed only slight variations for IL-6 and IL-8 with respect to the control cultures (data not shown).

In summary, we found a general stimulatory potency of *p*-NP for GM-CSF, IFN-γ, IL-1β, IL-4, TNF-α, and IL-10 at extremely low *p*-NP concentrations, with a suppressive effect for TNF-α at 10^−13^ M *p*-NP. IL-2 and IL-5 were below the LOD in both *p*-NP–treated and control cultures. IL-6 and IL-8 were the most abundant cytokines, but their levels were not affected by *p*-NP treatment. We observed significant differences with respect to control cultures only for GM-CSF and IL-10 (*p* = 0.045 and *p* = 0.011, respectively). These results do not exclude possible differences for the other cytokines tested; they show only that the low number of samples containing detectable amounts of some cytokines and the high variability of individual measurements did not allow us to highlight the differences with a low probability of error.

### Detection of β-hCG

We analyzed β-hCG secretion, a marker of trophoblast viability, by a specific immunoenzymometric assay in parallel with the cytokine assays. We detected β-hCG in 100% of the samples, with no significant difference with respect to control cultures at any of the *p*-NP concentrations (data not shown).

## Discussion

Cytokines play a critical role in pregnancy, particularly in the early stages during blastocyst implantation and placental development (Chaouat et al. 2007; Moffett and Loke 2006; Paria et al. 2002; Saito 2001; Schäfer-Somi 2003). Therefore, it is extremely useful to study cytokines secreted by the placenta in the first trimester of pregnancy, as well as potential interfering factors.

Using an *in vitro* model of chorionic villous explants, we showed here that *p*-NP, a ubiquitous environmental contaminant with estrogenic activity, interfered with first-trimester human placenta cytokine secretion. In particular, we assayed a set of 10 cytokines (GM-CSF, IFN-γ, IL-1β, IL-2, IL-4, IL-5, IL-6, IL-8, IL-10, and TNF-α) in the culture medium of chorionic villous explants exposed to *p*-NP or only to vehicle (control). The findings revealed an increase of GM-CSF, IFN-γ, IL-1β, IL-4, and IL-10, with a maximum activity at 10^−9^ M *p*-NP for GM-CSF and 10^−11^ M *p*-NP for the other cytokines, with a significant difference for GM-CSF and IL-10 at 10^−11^ M *p*-NP. TNF-α release was also modulated by *p*-NP treatment with a decrease at 10^−13^ M and an increase at higher *p*-NP concentrations. IL-6 and IL-8, the most abundantly secreted cytokines, were not affected by *p*-NP treatment at any concentration, whereas IL-2 and IL-5 were below the detection limit in *p*-NP–treated and untreated placental explants. To demonstrate tissue integrity in our explant cultures, we verified that β-hCG secretion did not change significantly in *p*-NP–treated versus control cultures. β-hCG release is a marker of continuous endocrine activity of the syncytiotrophoblast, the epithelial layer of chorionic villi forming the placental barrier (Miller et al. 2005). Using the same model of placental explants, we previously showed that 10^−9^ M *p*-NP had an estrogen-like effect, in that it increased β-hCG release and cellular apoptosis at 48–72 hr of *p*-NP exposure (Bechi et al. 2006). In accordance with these previous findings, we found no remarkable changes in β-hCG release at 24 hr of exposure, even at 10^−9^ M *p*-NP, the highest concentration used. These data reveal that *p*-NP deregulates cytokine secretion before it acts on β-hCG release.

GM-CSF and IL-10, the two cytokines significantly affected by *p*-NP, are important mediators in human pregnancy (Gotsch et al. 2008; Robertson 2007). Indeed, GM-CSF is a pivotal factor contributing to normal placental development and fetal growth (Robertson 2007). It is expressed in early gestation by fetal trophoblast and by decidual immune and nonimmune cells (Jokhi et al. 1994; Saito et al. 1993). It also acts on placental trophoblast cells by inducing differentiation into mature syncytiotrophoblast and stimulates its secretion of placental lactogen and chorionic gonadotropin (Baldwin 1992; Garcia-Lloret et al. 1994; Morrish et al. 1998). A very recent report by Fukui et al. (2008) showed that GM-CSF-producing natural killer (NK) cells in the maternal decidua tended to be decreased in women with recurrent abortion or implantation failure. All these studies suggest that GM-CSF is a crucial autocrine/paracrine mediator at the maternal–fetal interface regulating placental growth and its acceptance in the maternal uterus.

IL-10 is an anti-inflammatory cytokine widely present in tissues and fluids during gestation (Gotsch et al. 2008), including cyto- and syncytiotrophoblast as well as decidual mononuclear cells/macrophages and NK cells (Hanna et al. 2000; Lidstrom et al. 2003). High levels of IL-10 have also been found in amniotic fluid and in the maternal serum (Dudley et al. 1997; Gotsch et al. 2008; Greig et al. 1995). IL-10 is one of the cytokines produced by T-helper (Th)2 lymphocytes, whose immunity is necessary for successful fetal outcome. During pregnancy, Th1-driven cell-mediated immunity characterized by the secretion of IFN-γ, TNF-α, and IL-2 is shifted to a Th2-driven humoral immunity mainly represented by the secretion of IL-4, IL-5, IL-6, and IL-10 (Wegmann et al. 1993). Deviation from the Th2-like response leads to miscarriage (Raghupathy and Kalinka 2008; Tezabwala et al. 1989), and the administration of Th1 cytokines in mice has also been shown to cause miscarriage (Tezabwala et al. 1989).

The present study shows that Th2 placental cytokines (IL-4 and IL-10) are increased by *p*-NP treatment, whereas Th1 cytokines are increased, as in the case of IFN-γ, or modulated by a decrease or increase depending on the *p*-NP concentration, as in the case of TNF-α. These findings agree with Lee et al. (2003) and Iwata et al. (2004) in showing an imbalance of Th1/Th2 cytokines in immune cells caused by *p*-NP exposure. Specifically, Lee et al. (2003) demonstrated that *p*-NP increased IL-4 production in CD4^+^ T cells and Iwata et al. (2004) showed that *p*-NP and octylphenol had direct effects on T cells, suppressing the Th1 and enhancing the Th2 cytokine development.

The consequences of this altered balance at the maternal–fetal interface are not known. Literature reports have shown that some of the cytokines increased in this study (GM-CSF, IL-4, and IL-10) are able to stimulate β-hCG secretion by the trophoblast cells (Doria et al. 2006; Nishino et al. 1990; Saito et al. 1997). These findings, along with ours, suggest that, by altering cytokine balance and therefore trophoblast differentiation, *p*-NP exposure in early gestation may contribute to faulty placentation, leading to pregnancy disorders and fetal growth retardation (Allaire et al. 2000; Crocker at al. 2004).

The *p*-NP concentrations used here are in the nano- to picomolar range, well below (< 1,000 times) those reported in other *in vitro* systems or those found in humans (Blom et al. 1998; Inoue et al. 2000; Kawaguchi et al. 2004; Nagel et al. 1997; Tan and Nohd 2003). To our knowledge, no biological effects have been reported for *p*-NP at concentrations < 1 μM, except for the recent finding of a dose-dependent inhibition of aromatase activity in human JEG-3 placenta cells in the range of 10^−9^ to 10^−5^ M (Bonefeld-Jørgensen et al. 2007). The present study supports the high sensitivity of human placenta to *p*-NP exposure. The most effective concentration (10^−11^ M) used in the present study is about 2.2 pg/mL, whereas the levels of *p*-NP detected in human samples vary from 0.3 to 221.7 ng/mL, and in the environment, 4.1 μg/L in river waters and 1 mg/kg in sediments (Inoue et al. 2000; Kawaguchi et al. 2004; Soares et al. 2008). Lower levels have been reported (15.17 ng/mL) in human cord blood samples (Tan and Nohd 2003).

## Conclusion

Our study demonstrates that *p*-NP at environmentally relevant levels affects the cytokine balance in human placenta. Environmental levels of *p*-NP may be well above the threshold necessary to induce paracrine disruption in human placenta. These results raise concern about maternal exposure to this chemical during pregnancy and suggest a possible involvement of environmental factors in pregnancy complications.

## Figures and Tables

**Table 1:** Cytokine concentration (pg/mg total tissue protein) in culture medium from *p*-NP–treated and –untreated chorionic villous explants.

Group	GM-CSF	IFN-γ	IL-1β	IL-2	IL-4	IL-5	IL-10	TNF-α	IL-6	IL-8
Control										
*n*	6	6	6	6	6	6	5	3	6	6
Mean (pg/mg)	0.5370	0.8438	2.7867	< LOD	1.9622	< LOD	0.1154	0.4214	> LOD	> LOD
SD	0.3251	0.7144	1.2905		1.0012		0.0965	0.2841		
Variance	0.1060	0.5100	1.6650		1.0020		0.0090	0.0810		

*p*-NP 10^−13^ M										
*n*	6	6	6	6	6	6	5	3	6	6
Mean (pg/mg)	0.5439	0.7426	2.9217	< LOD	2.2076	< LOD	0.0948	0.2806	> LOD	> LOD
SD	0.2215	0.4056	0.9331		0.7652		0.0423	0.1178		
Variance	0.0490	0.1650	0.8710		0.5850		0.0020	0.0140		

*p*-NP 10^−11^ M										
*n*	6	6	6	6	6	6	5	3	6	6
Mean (pg/mg)	0.7676	1.2933	3.3403	< LOD	2.7319	< LOD	0.1757	0.6541	> LOD	> LOD
SD	0.5036	1.0250	1.2457		1.5103		0.1570	0.8087		
Variance	0.2540	1.0510	1.5520		2.2810		0.0250	0.6540		

*p*-NP 10^−9^ M										
*n*	6	6	6	6	6	6	5	3	6	6
Mean (pg/mg)	0.7337	1.0893	3.3070	< LOD	2.6281	< LOD	0.1904	0.6708	> LOD	> LOD
SD	0.4666	0.8686	1.7310		1.8578		0.2024	0.4574		
Variance	0.2180	0.7540	2.9960		3.4510		0.0410	0.2090		

< LOD and > LOD indicate values outside the detection limit.

## References

[b1-ehp-118-427] Allaire AD, Ballenger KA, Wells SR, Lessey BA (2000). Placental apoptosis in pre-eclampsia. Obstet Gynecol.

[b2-ehp-118-427] Baldwin GC (1992). The biology of granulocyte-macrophage colony-stimulating factor: effect on hematopoietic and nonhematopoietic cells. Dev Biol.

[b3-ehp-118-427] Bechi N, Ietta F, Romagnoli R, Focardi S, Corsi I, Buffi C (2006). Estrogen-like response to *p*-nonylphenol in human first trimester placenta and BeWo choriocarcinoma cells. Toxicol Sci.

[b4-ehp-118-427] Blair RM, Fang H, Branham WS, Hass BS, Dial SL, Moland CL (2000). The estrogen receptor relative binding affinities of 188 natural and xenochemicals: structural diversity of ligands. Toxicol Sci.

[b5-ehp-118-427] Blom A, Ekman E, Johannisson A, Norrgren L, Pesonen M (1998). Effects of xenoestrogenic environmental pollutants on the proliferation of a human breast cancer cell line (MCF-7). Arch Environ Contam Toxicol.

[b6-ehp-118-427] Bonefeld-J⊘rgensen EC, Long M, Hofmeister MV, Vinggaard AM (2007). Endocrine disrupting potential of bisphenol A, bisphenol A dimethacrylate, 4-*n*-nonylphenol, and 4-*n*-octylphenol *in vitro*: new data. Environ Health Perspect.

[b7-ehp-118-427] Caniggia I, Taylor CV, Ritchie JW, Lye SJ, Letarte M (1997). Endoglin regulates trophoblast differentiation along the invasive pathway in human placental villous explants. Endocrinology.

[b8-ehp-118-427] Chaddha V, Viero S, Huppertz B, Kingdom J (2004). Developmental biology of the placenta and the origins of placental insufficiency. Semin Fetal Neonatal Med.

[b9-ehp-118-427] Chaouat G, Dubanchet S, Ledée N (2007). Cytokines: important for implantation?. J Assist Reprod Genet.

[b10-ehp-118-427] Chaouat G, Menu E, Clark DA, Dy M, Minkowski M, Wegmann TG (1990). Control of fetal survival in CBAxDBA/2 mice by lymphokine therapy. J Reprod Fertil.

[b11-ehp-118-427] Choi KC, Leung PC, Jeung EB (2005). Biology and physiology of calbindin-D9k in female reproductive tissues: involvement of steroids and endocrine disruptors. Reprod Biol Endocrinol.

[b12-ehp-118-427] Crocker IP, Tansinda DM, Baker PN (2004). Altered cell kinetics in cultured placental villous explants in pregnancies complicated by pre-eclampsia and intrauterine growth restriction. J Pathol.

[b13-ehp-118-427] Cross JC, Werb Z, Fisher SJ (1994). Implantation and the placenta: key pieces of the development puzzle. Science.

[b14-ehp-118-427] de Weert J, de la Cal A, van den Berg H, Murk A, Langenhoff A, Rijnaarts H (2008). Bioavailability and biodegradation of nonylphenol in sediment determined with chemical and bioanalysis. Environ Toxicol Chem.

[b15-ehp-118-427] Doria A, Iaccarino L, Sarzi-Puttini P, Ghirardello A, Zampieri S, Arienti S (2006). Estrogens in pregnancy and systemic lupus erythematosus. Ann NY Acad Sci.

[b16-ehp-118-427] Dudley DJ, Hunter C, Mitchell MD, Varner MW (1997). Amniotic fluid interleukin-10 (IL-10) concentrations during pregnancy and with labor. J Reprod Immunol.

[b17-ehp-118-427] Fujimoto J, Nakagawa Y, Toyoki H, Sakaguchi H, Sato E, Tamaya T (2005). Estrogen-related receptor expression in placenta throughout gestation. J Steroid Biochem Mol Biol.

[b18-ehp-118-427] Fukui A, Kwak-Kim J, Ntrivalas E, Gilman-Sachs A, Lee SK, Beaman K (2008). Intracellular cytokine expression of peripheral blood natural killer cell subsets in women with recurrent spontaneous abortions and implantation failures. Fertil Steril.

[b19-ehp-118-427] Garcia-Lloret MI, Morrish DW, Wegmann TG, Honore L, Turner AR, Guilbert LJ (1994). Demonstration of functional cytokine-placental interactions: CSF-1 and GM-CSF stimulate human cytotrophoblast differentiation and peptide hormone secretion. Exp Cell Res.

[b20-ehp-118-427] Gotsch F, Romero R, Kusanovis JP, Erez O, Espinoza J, Kim CJ (2008). The anti-inflammatory limb of the immune response in preterm labor, intra-amniotic infection/inflammation, and spontaneous parturition at term: a role of interleukin-10. J Matern Fetal Neonatal Med.

[b21-ehp-118-427] Greig PC, Herbert WN, Robinette BL, Teot LA (1995). Amniotic fluid interleukin-10 (IL-10) concentrations increase through pregnancy and are elevated in patients with preterm labor associated with intrauterine infection. Am J Obstet Gynecol.

[b22-ehp-118-427] Guenther K, Heinke V, Thiele B, Kleist E, Prast H, Raecker T (2003). Endocrine disrupting nonylphenols are ubiquitous in food. Environ Sci Technol.

[b23-ehp-118-427] Hanna N, Hanna I, Hleb M, Wagner E, Dougherty J, Balkundi D (2000). Gestational age-dependent expression of IL-10 and its receptor in human placental tissues and isolated cytotrophoblasts. J Immunol.

[b24-ehp-118-427] Hong EJ, Choi KC, Jeung EB (2004a). Induction of calbindin-D9K messenger RNA and protein by maternal exposure to alkylphenols during late pregnancy in maternal and neonatal uteri of rats. Biol Reprod.

[b25-ehp-118-427] Hong EJ, Choi KC, Jung YW, Leung PC, Jeung EB (2004b). Transfer of maternally injected endocrine disruptors through breast milk during lactation induces neonatal calbindin-D9k in the rat model. Reprod Toxicol.

[b26-ehp-118-427] Inoue K, Yoshimura Y, Makino T, Nakazawa H (2000). Determination of 4-nonylphenol and 4-octylphenol in human blood samples by high performance liquid chromatography with multi-electrode electrochemical coulometric-array detection. Analyst.

[b27-ehp-118-427] Iwata M, Eshima Y, Kagechika H, Miyaura H (2004). The endocrine disruptors nonylphenol and octylphenol exert direct effect on T cells to suppress Th1 development and enhance Th2 development. Immunol Lett.

[b28-ehp-118-427] Jokhi PP, King A, Loke YW (1994). Production of granulocyte-macrophage colony-stimulating factor by human trophoblast cells and by decidual large granular lymphocytes. Hum Reprod.

[b29-ehp-118-427] Kavlock RJ, Ankley GT (1996). A perspective on the risk assessment process for endocrine-disruptive effects on wildlife and human health. Risk Anal.

[b30-ehp-118-427] Kawaguchi M, Inoue K, Sakui N, Ito R, Izumi S, Makino T (2004). Stir bar sorptive extraction and thermal desorption-gas chromatography-mass spectrometry for the measurement of 4-nonylphenol and 4-tert-octylphenol in human biological samples. J Chromatogr B Analyt Technol Biomed Life Sci.

[b31-ehp-118-427] Kwack SJ, Kwon O, Kim HS, Kim SS, Kim SH, Sohn KH (2002). Comparative evaluation of alkylphenolic compounds on estrogenic activity *in vitro* and *in vivo*. J Toxicol Environ Health A.

[b32-ehp-118-427] Laird SM, Tuckerman EM, Li TC (2006). Cytokine expression in the endometrium of women with implantation failure and recurrent miscarriage. Reprod Biomed Online.

[b33-ehp-118-427] Laws SC, Carey SA, Ferrell JM, Bodman GJ, Cooper RL (2000). Estrogenic activity of octylphenol, nonylphenol, bisphenol A and methoxychlor in rats. Toxicol Sci.

[b34-ehp-118-427] Lee MH, Chung SW, Kang BY, Lee CH, Hwang SY, Kim TS (2003). Enhanced interleukin-4 production in CD4^+^ T cells and elevated immunoglobulin E levels in antigen-primed mice by bisphenol A and nonylphenol, endocrine disruptors: involvement of nuclear factor-AT and Ca^2+^. Immunology.

[b35-ehp-118-427] Lee MH, Kim E, Kim TS (2004). Exposure to 4-*ter*-octylphenol, an environmentally persistent alkylphenol, enhances interleukin-4 production in T cells via NF-AT activation. Toxicol Appl Pharmacol.

[b36-ehp-118-427] Lidstrom C, Matthiesen L, Berg G, Sharma S, Ernerudh J, Ekerfelt C (2003). Cytokine secretion patterns of NK cells and macrophages in early human pregnancy decidua and blood. Implications for suppressor macrophage in decidua. Am J Reprod Immunol.

[b37-ehp-118-427] Makrigiannakis A, Minas V (2007). Mechanisms of implantation. Reprod Biomed Online.

[b38-ehp-118-427] Miller RK, Genbacev O, Turner MA, Aplin JD, Caniggia I, Huppertz B (2005). Human placental explants in culture: approaches and assessments. Placenta.

[b39-ehp-118-427] Moffett A, Loke C (2006). Implantation, embryo-maternal interactions, immunology and modulation of the uterine environment—a workshop report. Placenta.

[b40-ehp-118-427] Monteiro-Riviere NA, Van Miller JP, Simon G, Joiner RL, Brooks JD, Riviere JE (2000). Comparative in vitro percutaneous absorption of nonylphenol and nonylphenol ethoxylates (NPE-4 and NPE-9) through human, porcine and rat skin. Toxicol Ind Health.

[b41-ehp-118-427] Morrish DW, Dakour J, Li H (1998). Functional regulation of human trophoblast differentiation. J Reprod Immunol.

[b42-ehp-118-427] Müller S, Schlatter C (1998). Natural and anthropogenic environmental oestrogens: the scientific basis for risk assessment. Oestrogenic potency of nonylphenol *in vivo*—a case study to evaluate the relevance of human non-occupational exposure. Pure Appl Chem.

[b43-ehp-118-427] Nagel SC, vom Saal FS, Thayer KA, Dhar MG, Boechler M, Welshons WV (1997). Relative binding affinity-serum modified access (RBA-SMA) assay predicts the relative *in vivo* bioactivity of the xenoestrogens bisphenol A and octylphenol. Environ Health Perspect.

[b44-ehp-118-427] Neubert D (1997). Vulnerability of the endocrine system to xenobiotic influence. Regul Toxico Pharmacol.

[b45-ehp-118-427] Nishino E, Matsuzaki N, Masuhiro K, Kameda T, Taniguchi T, Takagi T (1990). Trophoblast-derived interleukin-6 (IL-6) regulates human chorionic gonadotropin release through IL-6 receptor on human trophoblasts. J Clin Endocrinol Metab.

[b46-ehp-118-427] Paria BC, Reese J, Das SK, Dey SK (2002). Deciphering the cross-talk of implantation: advances and challenges. Science.

[b47-ehp-118-427] Raghupathy R, Kalinka J (2008). Cytokine imbalance in pregnancy complications and its modulation. Front Biosci.

[b48-ehp-118-427] Rama S, Petrusz P, Rao AJ (2004). Hormonal regulation of human trophoblast differentiation: a possible role for 17β-estradiol and GnRH. Mol Cell Endocrinol.

[b49-ehp-118-427] Robertson SA (2007). GM-CSF regulation of embryo development and pregnancy. Cytokine Growth Factor Rev.

[b50-ehp-118-427] Robertson SA, Mau VJ, Hudson SN, Tremellen KP (1997). Cytokine-leukocyte networks and the establishment of pregnancy. Am J Reprod Immunol.

[b51-ehp-118-427] Rudel RA, Camann DE, Spengler JD, Korn LR, Brody JG (2003). Phthalates, alkylphenols, pesticides, polybrominated diphenyl ethers, and other endocrine-disrupting compounds in indoor air and dust. Environ Sci Technol.

[b52-ehp-118-427] Saito S (2001). Cytokine cross-talk between mother and the embryo/placenta. J Reprod Immunol.

[b53-ehp-118-427] Saito S, Harada N, Ishii N, Morii T, Sakakura S, Enomoto M (1997). Functional expression on human trophoblasts of interleukin 4 and interleukin 7 receptor complexes with a common gamma chain. Biochem Biophys Res Commun.

[b54-ehp-118-427] Saito S, Nishikawa K, Morii T, Enomoto M, Narita M, Motoyoshi K (1993). Cytokine production by CD16-CD56 bright natural killer cells in the human early pregnancy decidua. Int Immunol.

[b55-ehp-118-427] Schäfer-Somi S (2003). Cytokines during early pregnancy of mammals: a review. Anim Reprod Sci.

[b56-ehp-118-427] Soares A, Guiyesse B, Jefferson E, Cartmell E, Lester JN (2008). Nonylphenol in the environment: a critical review on occurrence, fate, toxicity and treatment in wastewaters. Environ Int.

[b57-ehp-118-427] Sonnenschein C, Soto AM (1998). An update review of environmental estrogen and androgen mimics and antagonists. J Steroid Biochem Mol Biol.

[b58-ehp-118-427] Soto AM, Justicia H, Wray JW, Sonnenschein C (1991). *p*-Nonylphenol: an estrogenic xenobiotic released from “modified” polystyrene. Environ Health Perspect.

[b59-ehp-118-427] Soto AM, Sonnenschein C, Chung KL, Fernandez MF, Olea N, Serrano FO (1995). The E-SCREEN assay as a tool to identify estrogens: an update on estrogenic environmental pollutants. Environ Health Perspect.

[b60-ehp-118-427] Srivastava MD, Lippes J, Srivastava BI (1996). Cytokines of the human reproductive tract. Am J Reprod Immunol.

[b61-ehp-118-427] Tan BLL, Nohd MA (2003). Analysis of selected pesticides and alkylphenols in human cord blood by gas chromatography-mass spectrometer. Talanta.

[b62-ehp-118-427] Ter Veld MG, Zawadzka E, van den Berg JH, van der Saag PT, Rietjens IM, Murk AJ (2008). Food-associated estrogenic compounds induce estrogen receptor-mediated luciferase gene expression in transgenic male mice. Chem Biol Interact.

[b63-ehp-118-427] Tezabwala BU, Johnson PM, Rees RC (1989). Inhibition of pregnancy viability in mice following IL-2 administration. Immunology.

[b64-ehp-118-427] Vilcek J, Le J, Thomson A (1994). Immunology of cytokines: an introduction. The Cytokine Handbook.

[b65-ehp-118-427] Wegmann TG, Lin H, Guilbert L, Mossman TR (1993). Bidirectional cytokines interactions in the materno fetal relationship: successful allopregnancy is Th2 phenomenon. Immunol Today.

[b66-ehp-118-427] White R, Jobling S, Hoare SA, Sumpter JP, Parker MG (1994). Environmentally persistent alkylphenolic compounds are estrogenic. Endocrinology.

